# Implications of Myocardial Fibrosis Burden on Left Ventricular Systolic Function in Sepsis Survivors: Insights from a Retrospective Cohort Study Using Quantitative Late Gadolinium Enhancement Cardiovascular Magnetic Resonance

**DOI:** 10.3390/jcdd12080306

**Published:** 2025-08-13

**Authors:** Shayan Datta, Samuel Malomo, Thomas Oswald, Claire Phillips, Barbara Philips, Joon Lee, David Hildick-Smith, Victoria Parish, Alexander Liu

**Affiliations:** 1 Sussex Cardiac Centre, Royal Sussex County Hospital, Brighton BN2 5BE, UK; s.datta1@nhs.net (S.D.); s.malomo@nhs.net (S.M.); david.hildick-smith@nhs.net (D.H.-S.); victoria.parish@nhs.net (V.P.); 2 Intensive Care Unit, Royal Sussex County Hospital, Brighton BN2 5BE, UKbarbara.philips@nhs.net (B.P.); 3 Clinical and Experimental Medicine, Brighton and Sussex Medical School, Brighton BN1 9PX, UK; 4 Department of Radiology, Royal Sussex County Hospital, Brighton BN2 5BE, UK; joon.lee@nhs.net

**Keywords:** sepsis, cardiomyopathy, heart failure, late gadolinium enhancement, cardiovascular magnetic resonance

## Abstract

**Background:** After recovery from acute sepsis, patients can exhibit left ventricular systolic dysfunction (LVSD) and non-ischaemic myocardial fibrosis. The relationship between myocardial fibrosis and LVSD remains poorly defined. This study sought to fill this knowledge gap using quantitative late gadolinium enhancement (LGE) cardiovascular magnetic resonance (CMR). **Methods:** Twenty-eight sepsis survivors underwent CMR at 1.5-Tesla for the assessment of cardiac volumes, systolic function and LGE. Myocardial fibrosis burden was derived quantitatively by LGE, expressed as a percentage of LV mass. **Results:** Study patients (age 51 ± 16 years; 57% males) had a median LVEF of 59% (IQR: 43–64) of whom 43% had LVSD (LV ejection fraction [LVEF] < 50%). LGE was found in 64% of the study patients by visual assessment, mostly in non-ischaemic patterns. The overall myocardial fibrosis burden was 3.3% (IQR: 0.9–7.1) of LV mass. Myocardial fibrosis burden was inversely correlated to LVEF in sepsis survivors (Rho = -0.385; *p* = 0.043). Patients with LVSD had greater myocardial fibrosis burden than patients without LVSD (7.3 ± 6.0% vs. 3.1 ± 2.5%; *p* = 0.041). Myocardial fibrosis burden was not significantly influenced by the presence of major co-morbidities. **Conclusions:** Myocardial fibrosis burden may play a role in LV dysfunction in sepsis survivors. Further work is needed to better understand its prognostic value.

## 1. Introduction

Sepsis is a life-threatening, multi-systemic inflammatory response to infection [1,2]. It remains one of the most prolific causes of mortality worldwide, despite improvements in the recognition of acute sepsis and prompt antibiotic therapy [3,4,5,6]. Cardiac dysfunction can be observed in patients during the acute septic episode, which is traditionally believed to be a reversible condition [7,8,9,10,11]. Left ventricular (LV) systolic failure can occur in about a quarter of patients during acute sepsis which may be related to the acute inflammatory response and endothelial dysfunction [10,12]. The precise interplay between myocardial contractile failure and inflammatory changes during sepsis remains incompletely understood [5]. The management of this condition predominantly hinges on timely treatment of the underlying infection and organ support [5].

Whilst acute sepsis has a significant mortality risk associated with it, survivors of sepsis are also extremely prevalent worldwide [13,14,15,16]. These patients are at an increased risk of re-hospitalisation or may suffer prolonged, often debilitating, multi-systemic symptoms, leading to significant morbidity and reduced quality of life [13,14,15,16]. The healthcare cost of managing sepsis on a global scale is in the order of multiple billions of dollars each year [17]. Sepsis survivors also have an elevated risk of developing long term cardiovascular complications, such as myocardial infarction, strokes, heart failure and increased mortality [3]. The underlying mechanism for this prognostic observation is currently unclear.

Recent evidence showed that in sepsis survivors, i.e., patients who have recovered from their acute septic episode, left ventricular (LV) dilatation, LV dysfunction and non-ischaemic patterns of myocardial fibrosis can still be present [18,19]. These patients did not have a known pre-existing clinical diagnosis of heart failure [18], suggesting that both structural and functional abnormalities may persist beyond the initial septic response [18]. This pattern of post-sepsis cardiac abnormalities seems to correlate with previous autopsy reports of macroscopic cardiac dilatation, microscopic myocardial necrosis and fibrosis in sepsis patients [20]. It remains unclear whether LV systolic dysfunction (LVSD) in sepsis survivors is related to the presence of myocardial fibrosis. This would be an important question to answer in the path to deciphering the underlying mechanism for heart failure in sepsis survivors.

Cardiovascular magnetic resonance (CMR) imaging provides multi-parametric and non-invasive assessment of cardiac structure, function and tissue characterisation [21,22,23,24]. CMR is considered a reference standard method for the evaluation of cardiac biventricular volumes and systolic function [21]. Late gadolinium enhancement (LGE) imaging also provides a detailed assessment of myocardial infarction and the delineation of non-ischaemic patterns of focal myocardial fibrosis [21]. LGE images can be post-processed to express the myocardial fibrosis as a percentage of total myocardial mass, which provides a quantitative evaluation of myocardial scar burden in a range of cardiomyopathies [25]. Additionally, CMR plays a key role in distinguishing transient left ventricular dysfunction (TLVD) due to myocardial stunning or hibernation from irreversible injury [24,26]. T2-weighted imaging can identify myocardial oedema, indicative of acute or reversible injury, while LGE differentiates viable from non-viable myocardium based on the presence and pattern of scarring [24,26].

In this study, we aimed to assess the burden of myocardial fibrosis in sepsis survivors using quantitively LGE analysis by CMR. We also investigated the relationship between the burden of myocardial fibrosis and left ventricular systolic function in this patient cohort.

## 2. Materials and Methods

### 2.1. Study Subjects

Consecutive adult sepsis survivors (≥18 years old) treated at the University Hospitals Sussex National Health Service (NHS) Foundation Trust (UK) between June 2018 and April 2025 who subsequently underwent clinical cardiovascular magnetic resonance (CMR) were retrospectively included in the study. Patients underwent CMR due to either the presence of cardiac abnormalities on echocardiography or the presence of cardiac symptoms. The CMR scans were performed at the Royal Sussex County Hospital, Brighton (a UK tertiary cardiac centre). Patients were excluded if they did not tolerate the CMR scan (*n* = 1). A total of 28 patients were included in the study.

### 2.2. Ethical Approval Statement

This retrospective study was approved by the Research and Innovation department of the University Hospitals Sussex NHS Foundation Trust and informed patient consent was waived.

### 2.3. Clinical Data Collection

Clinical data of the study patients were collected from the electronic hospital records. These included demographic data, cardiac symptoms, cardiac past medical history and medications. To ensure accuracy, the data were validated by a second observer against the source electronic hospital records.

### 2.4. CMR

Patients underwent CMR at 1.5 Tesla using a standard clinical protocol including cine and LGE imaging, as previously described [21,22]. Fourteen patients underwent CMR using a Siemens scanner (Aera, Siemens Healthineers, Erlangen, Germany). In August 2023, a new MRI centre was opened in our hospital, and the remaining 14 study patients underwent CMR using a Philips scanner (Ingenia Ambition, Philips Healthcare, Best, The Netherlands).

Cine images were acquired in both long-axis and short-axis planes using a balanced steady-state-free-precession sequence as previously described [21]. LGE images were acquired in matching long- and short-axis planes to cines, approximately 6–8 min after an intravenous bolus of gadolinium-based contrast agent (0.1 mmol/kg), (Dotarem, Guerbet, France), followed by a saline flush [18].

### 2.5. CMR Image Analysis

Cardiac ventricular volumes and systolic function were analysed using commercially available software (cvi42, Circle Cardiovascular Imaging, Calgary, Canada) by experienced CMR consultants. LGE images were qualitatively assessed visually by the same CMR consultants, noting the location and extent of enhancement.

Quantitative LGE image analysis was also performed using cvi42 (Circle Cardiovascular Imaging, Calgary, Canada), as previously described [27]. Endocardial and epicardial borders were manually traced before a region of interest (ROI) was placed in the remote (unenhanced) myocardium. Regions of myocardial fibrosis were defined as pixels with signal intensity 5 standard deviations above the mean signal intensity of the remote myocardial ROI [28]. Where possible the remote myocardial ROI was placed on the contralateral wall of the region of myocardial fibrosis. The burden of myocardial fibrosis was defined as the total enhanced myocardial mass expressed as a percentage of the total LV mass.

### 2.6. Statistical Analysis

Parametric data were expressed as mean ± standard deviation (SD). Non-parametric data were expressed as median (interquartile range; IQR) Two groups of parametric data were compared using the independent sample *t*-test. Non-parametric data were compared using the Mann–Whitney test. Relationships between two continuous variables were assessed using Spearman’s rank correlation coefficient (Rho). Agreement in the analysis performed between the two independent observers was assessed using the Bland–Altman method. Correlation between the analysis performed by the two independent observers was also estimated using the intraclass correlation coefficient (ICC), with 95% confidence intervals (CI). *p* values < 0.05 denote statistical significance. Statistical analysis was performed using commercially available software (MedCalc, version 20.104, Mariakerke, Belgium) and were validated by a second observer to ensure accuracy.

## 3. Results

### 3.1. Patient Clinical Data

Sepsis survivors (mean age 51 ± 16 years old; 57% males) had a range of cause of sepsis, including respiratory (64%), gastrointestinal (7%), abscess (7%) and sepsis of unknown origin (11%; Table 1). Half of the patients required treatment in intensive care units (Table 1). Patients had elevated C-reactive protein (CRP), white cell counts (WCC) and high-sensitivity cardiac troponin levels (hs-cTn; Table 1). The commonest cardiac sounding symptom was dyspnoea (39%), followed by chest pain (25%) and palpitations (14%; Table 1).

The clinical characteristics of the study patients are shown in Table 2. Atrial fibrillation (AF; 21%), hypertension (18%) and smoking (18% current/ex-smoking) were amongst the commonest co-morbidities (Table 2). Post-sepsis, half of patients were taking beta-blockers. A significant proportion of sepsis survivors were also taking other medications, such as mineralocorticoid receptor blockers (MRA; 43%), Sodium-glucose co-transporter-2 (SGLT-2) inhibitors (39%), angiotensin-converting enzyme inhibitor/angiotensin receptor blocker (ACEi/ARB; 18%) and sacubitril/valsartan (25%).

### 3.2. CMR Parameters in Sepsis Survivors

The CMR scans were performed in sepsis survivors a median of 65 days (IQR 12–124) after their acute sepsis episode (Table 3). The overall LV ejection fraction (LVEF) in the study patient cohort was 59% (43–64) and the overall RV ejection fraction (RVEF) was 53% (48–60). Of the study patients, 43% had evidence of LVSD by CMR (defined as LVEF < 50% [29]) and 46% had evidence LV cavity dilatation.

Evidence of LGE was found in 18 out of 28 (64%) of the study patients by qualitative visual assessment, most of which were in a non-ischaemic pattern (16/18 cases; Table 3). The overall burden of myocardial fibrosis by quantitative LGE analysis was 3.3% (0.9–7.1) of LV mass (Table 3). No RV LGE was observed in the study patients.

### 3.3. Relationship Between Myocardial Fibrosis and LV Systolic Function in Sepsis Survivors

**Figure 1:**
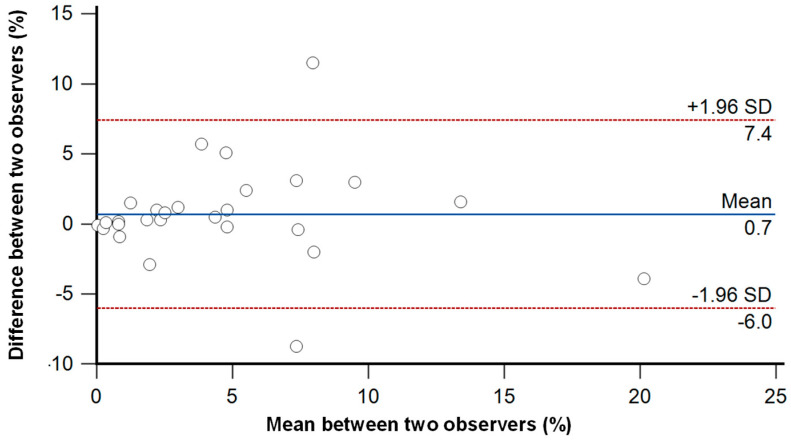
Bland-Altman plot for the inter-observer variability between 2 independent observers for quantitative LGE analysis.

Quantitative LGE analysis was performed by two independent observers, showing good agreement between the values obtained (Figure 1). The ICC between the two independent observers was 0.86 (95% CI 0.70–0.93).

**Figure 2:**
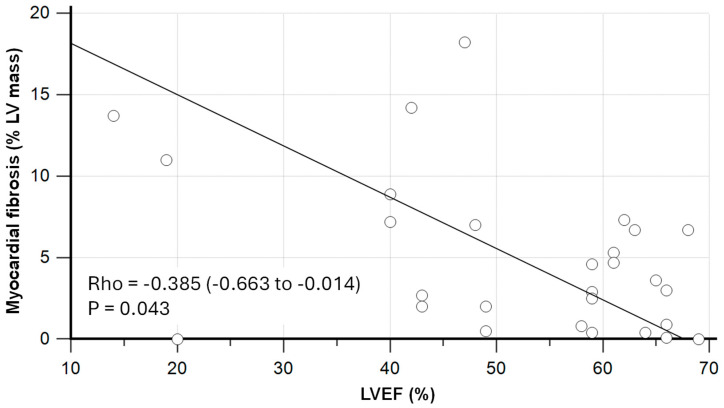
Relationship between myocardial fibrosis and left ventricular ejection fraction (LVEF) in sepsis survivors as assessed by cardiovascular magnetic resonance (CMR). Rho was estimated using Spearman’s rank correlation coefficient, displayed with 95% confidence interval.

The burden of myocardial fibrosis, as assessed by quantitative LGE (expressed as % of LV mass), was inversely correlated to the LVEF in sepsis survivors (Spearman’s Rho = -0.385; *p* = 0.043; Figure 2).

**Figure 3:**
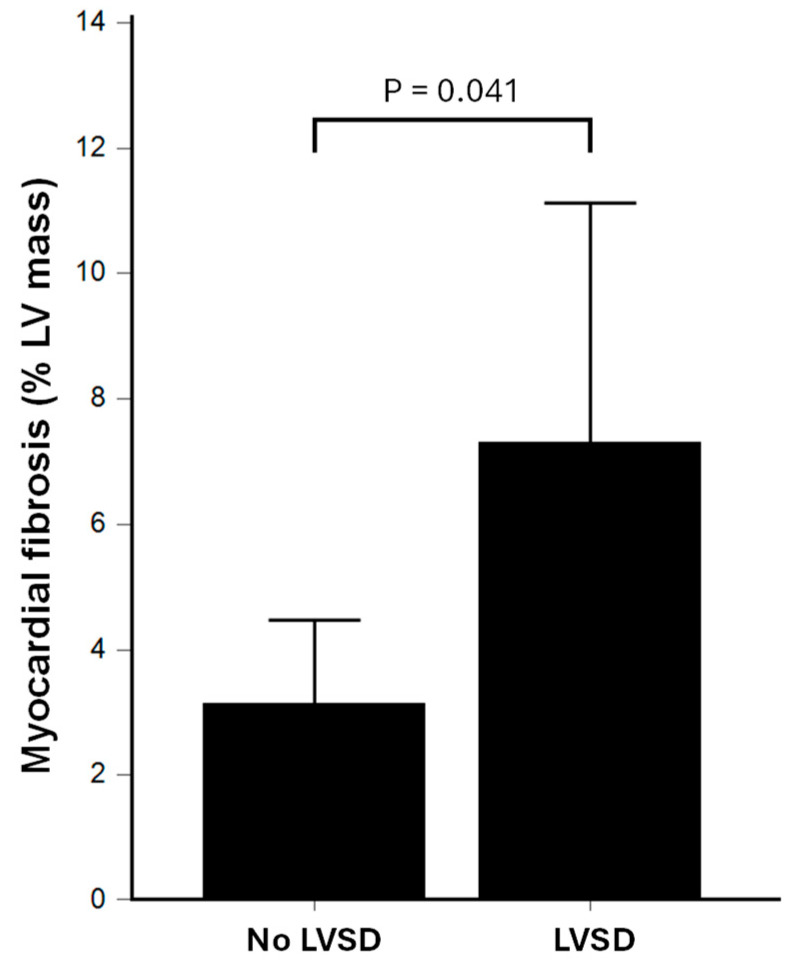
Comparison of myocardial fibrosis burden as assessed by quantitative LGE between sepsis survivors with left ventricular systolic dysfunction (LVSD) vs. patients without LVSD. LGE: late gadolinium enhancement.

Patients with LVSD (LVEF < 50%; *n* = 12), had significantly greater myocardial fibrosis burden than patients with preserved LV systolic function (LVEF ≥ 50%; *n* = 16), as assessed by quantitative LGE (7.3 ± 6.0% vs. 3.1 ± 2.5%; *p* = 0.041; Figure 3). Similarly, patients with positive LGE had significantly lower LVEF as compared to patients with negative LGE (48 ± 17% vs. 60 ± 7%; *p* = 0.017). 

In the study patients, myocardial fibrosis burden was not significantly correlated to LV end-diastolic volume index (LVEDVi; Rho = 0.273; *p* = 0.160), LV end-systolic volume index (LVESVi; Rho = 0.341; *p* = 0.075) or LV stroke volume index (LVSVi; Rho = -0.248; *p* = 0.204).

### 3.4. Relationships Between Co-Morbidities and Myocardial Fibrosis in Sepsis Survivors

When stratified by cardiac comorbidities, myocardial fibrosis burden was not significantly different between patients with vs. without AF, hypertension, diabetes mellitus or chronic kidney disease (Table 4). Patients without hypercholesterolaemia paradoxically had greater myocardial fibrosis burden than patients with the condition (Table 4). When the co-morbidities were combined into a composite (one or more of each co-morbidity), patients with co-morbidities had similar myocardial fibrosis burden compared to patients without co-morbidities (Table 4).

**Figure 4:**
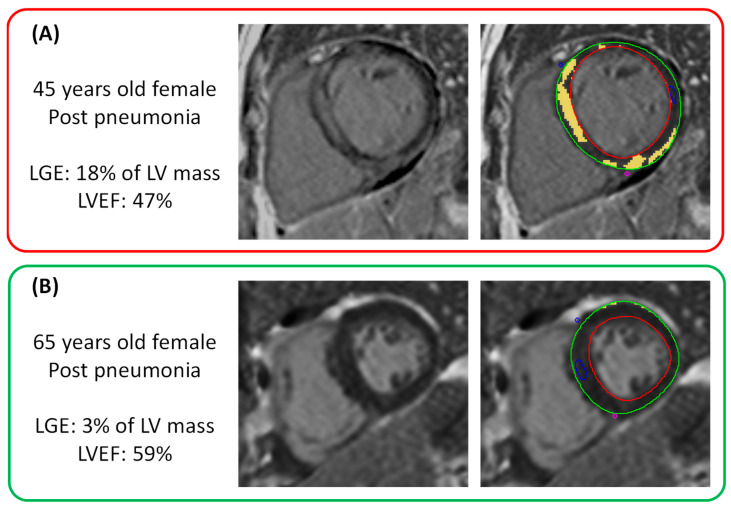
Illustrative examples of sepsis survivors with (Panel **A**) and without (Panel **B**) significant late gadolinium enhancement (LGE) on visual and quantitative analysis. Enhanced myocardium (yellow) is highlighted as regions with signal intensity 5 standard deviations above the mean signal intensity of remote myocardium (blue region of interest). LVEF: left ventricular ejection fraction.

Figure 4 shows illustrative examples of patients with and without significant late gadolinium enhancement on CMR.

## 4. Discussion

This study assessed the relationship between myocardial fibrosis burden and LV systolic function in sepsis survivors, using quantitative LGE analysis. The main findings are: (i) There was a weak inverse correlation between myocardial fibrosis burden and LVEF in sepsis survivors; (ii) patients with LVSD have a greater burden of myocardial fibrosis than those without LVSD; (iii) in this study patient cohort, myocardial fibrosis burden was not significantly affected by major co-morbidities; and (iv) myocardial fibrosis burden was not significantly related to individual indices of LV volumes (end-systolic, end-diastolic and stroke volume indices).

The findings suggest that myocardial fibrosis burden may play a role in the development of LV dysfunction in sepsis survivors. Further work is needed to assess the prognostic value of quantitative LGE analysis in post-sepsis patients and whether it can facilitate the development of novel therapies.

### 4.1. Relationship Between Myocardial Fibrosis and LV Dysfunction

Recent evidence suggested the presence of LVSD in patients who have recovered from acute sepsis [18]. Preliminary data also suggest that this LVSD may be responsive to guideline-directed medical therapy for heart failure in a small sample size [19]. It is not thought likely that LVSD post-sepsis can be fully explained by obstructive coronary disease [30]. Therefore, the LV dysfunction observed in sepsis survivors may have a non-ischaemic aetiology, which currently remains poorly characterised [18].

In a range of non-sepsis-related cardiomyopathies [31], and in ischaemic heart disease [32], the myocardial scar/fibrosis burden is related to the degree of LVSD. However, LV dysfunction can also occur in the absence of LGE [32]. LGE detects the presence of focal fibrosis by comparing regions of enhancement against regions of unenhanced myocardium [27,32]. LGE cannot be used to detect diffuse or interstitial myocardial fibrosis or changes in myocardial water content, such as those observed in myocardial oedema and inflammation [22,27,31]. This inherent limitation of an otherwise powerful technique in LGE means that a direct correlation between myocardial fibrosis burden (as detected by LGE) and LV systolic function would not likely be linear.

In this study, the LGE mass (expressed as a percentage of LV mass) was weakly correlated to LVEF, which suggests that, to some extent, the greater the burden of focal myocardial fibrosis, the worse the LV systolic function was likely to be. There were some patients who had LVSD but a relatively low burden of LGE by CMR. Conversely, a few patients had a relatively high burden of LGE but with preserved LV systolic function. These outliers support the notion that LV dysfunction in sepsis survivors is governed by factors beyond LGE-detectable focal myocardial fibrosis.

Indeed, myocardial inflammation and oedema are known to be present in several cardiomyopathies, such as dilated cardiomyopathy and cardiac sarcoidosis [33,34]. The relationship between myocardial oedema and LV dysfunction was not assessed in this study owing to the lack of data in the included clinical CMR scans. This research question deserves further investigation. The relationship between diffuse interstitial fibrosis and LV function is another important factor to further investigate in the post-sepsis patient population [35,36,37].

The results of this study suggest that myocardial fibrosis is likely one of several factors in the development of LVSD post-sepsis. Whether myocardial fibrosis burden portends a prognostic value in sepsis survivors needs to be tested in future studies.

### 4.2. Post-Sepsis Cardiomyopathy as a Disease Entity

Sepsis and its relationship with cardiac dysfunction propagate as part of a continuum in the disease progression. Based on current literature, cardiac dysfunction can be observed at two broad time points. The first time point is during the acute sepsis episode, when the patient is suffering from the acute phase inflammation, the systemic mal-adaptations including (but not limited to) vascular changes and the alterations in cardiac loading conditions [38]. This “acute sepsis cardiomyopathy” is the one that is often thought to be reversible [7,8]. Indeed, the reversibility of this acute sepsis cardiac dysfunction has been documented both in animal studies and in humans—it is a condition that has been studied for several decades with limited therapeutic breakthroughs [9,11,39,40,41]. The second time point where cardiac dysfunction can be observed is after the patient recovers from the acute septic episode [18]. This is the subject of this current study and other research efforts in a more contemporary field [18,19,30]. Indeed, the existence of a post-sepsis cardiomyopathy directly challenges the traditional paradigm that acute sepsis cardiac dysfunction is reversible.

The observation that myocardial fibrosis is present in sepsis survivors suggests that irreversible myocardial injury took place at some point during the acute/peri-sepsis period. This irreversible injury, along with the observation of concurrent LV dysfunction, would further challenge the possibility of cardiac functional recovery, if in the absence of dedicated medical therapy [19]. The results of this current study add further quantitative analysis of the burden of myocardial fibrosis using CMR and its possible interplay with LV dysfunction in sepsis survivors.

Whether this post-sepsis LV dysfunction developed *de novo* from the sepsis, or whether the LV dysfunction was pre-existing (i.e., present before sepsis) and that the sepsis episode simply “announced” these patients to medical services to make the diagnosis of LVSD, remains unclear. The closest we have come to ensuring that these patients did not have cardiac dysfunction before sepsis is by selecting patients who did not have any pre-existing (pre-sepsis) history of heart failure. Notably, the patients in the study also had a limited number of co-morbidities, further minimising the likelihood of there being pre-existing LV dysfunction.

To truly answer the question of whether the LV dysfunction post-sepsis is *de novo* would likely require population-based studies where large numbers of subjects are assessed with cardiac imaging at baseline, then followed up for many years to see which patients develop sepsis and undergo follow-up cardiac imaging. This study would likely depend on multi-centre collaborations and significant resource input. However, the possible results/outcomes from such a study could be a landmark in defining the aetiology and natural history of post-sepsis cardiomyopathy, to potentially open new doors for the development of novel therapeutic targets [42].

### 4.3. Limitations and Future Directions

This retrospective study was performed using a relatively small number of patients, with a limitation of being prone to sampling bias. Formal power calculation in this context was also less meaningful. A larger study is required to assess the prognostic value of quantitative LGE analysis in sepsis survivors. This study would also enable the assessment of the clinical workflow viability of performing quantitative LGE post-processing in this patient cohort. This study did not include data on myocardial oedema and inflammation imaging, which would be useful to further assess the contribution of these disease states to LV dysfunction. The lack of myocardial biopsy data, which is difficult to justify in this patient cohort, could also have enabled correlation with the LGE data. The lack of routine T1-/T2-mapping performance in the clinical CMR scans meant that meaningful assessment of the contribution of diffuse myocardial fibrosis to LV dysfunction was not possible in this study. This would be important to include in future studies. This study also did not have serial interval CMR studies, which would be helpful to assess the progression of the myocardial fibrosis and its relationship with LV function. Potential confounding variables, such as time-to-scan, ICU stay, peak hs-cTn, CKMB, CRP, medical therapy were not included in the correlation or group comparisons owing to the sample size. The effect of these factors should be investigated in future studies. We could not find any sepsis survivors without symptoms to act as controls in the study, since these patients would naturally have little indication to undergo a clinical CMR. Future studies which include asymptomatic sepsis survivors would provide an important comparison. Owing to the lack of availability of the feature tracking (FT) module at the time of this study, FT-analysis could not be performed. Future studies which investigate the relationship between CMR derived strain parameters and myocardial fibrosis would further enrich our understanding of the pathophysiology of sepsis survivors. Overall, despite the limitations, this first study to use quantitative LGE to correlate with LVEF in sepsis survivors does shed light on the potential usefulness of such method in this patient cohort.

## 5. Conclusions

This study suggests that myocardial fibrosis burden may contribute to LV dysfunction in sepsis survivors. Further research with larger cohorts, stricter inclusion criteria and strain imaging alongside LGE is needed to clarify the clinical implications in managing sepsis survivors. The prognostic value of quantitative LGE burden in sepsis survivors is also important to further evaluate.

## Figures and Tables

**Table 1:** Clinical features of the acute sepsis episode and post-sepsis symptoms.

	Patients (*n* = 28)
Age, years	51 ± 16
Male	16 (57)
BMI, kg/m^2^	25 ± 6
BSA, m^2^	1.9 ± 0.3
Sepsis cause	
Respiratory	18 (64)
Gastrointestinal	2 (7)
Abscess	2 (7)
Unknown origin	3 (11)
Other	3 (11)
Care escalation	
ICU	14 (50)
HDU/CCU	4 (14)
Ward-based care	10 (36)
Serum biomarkers	
Peak CRP, mg/L	256 ± 145 (*n* = 23)
Peak WCC, ×10^9^/L	17.5 (10.1–25.4) (*n* = 23)
Peak Hs-cTnT, ng/L	177 (39–532) (*n* = 18)
Post-sepsis symptoms	
Chest pain	7 (25)
Palpitations	4 (14)
Dyspnoea	11 (39)
Pre-syncope/ Syncope	1 (4)

BMI: body mass index; BSA: body surface area; CCU: cardiac care unit; CRP: C-reactive protein; HDU: high dependency unit; Hs-cTnT: high sensitivity cardiac troponin T; ICU: intensive care unit; and WCC: white cell count. Continuous variables are displayed as mean ± SD or median (interquartile range). Categorical variables were displayed as number (%).

**Table 2:** Clinical characteristics of post-sepsis patients.

	Patients (*n* = 28)
Comorbidities	
Hypertension	5 (18)
Diabetes mellitus	3 (11)
Smoking (ex- or current)	5 (18)
Hypercholesterolaemia	4 (14)
Atrial fibrillation	6 (21)
Ischaemic heart disease	2 (7)
Pre-sepsis heart failure	0 (0)
CKD	2 (7)
COPD/Asthma	5 (18)
Post-sepsis CV Medications	
Anti-platelet drugs	7 (25)
Beta-blocker	14 (50)
ACE-inhibitor/ARB	5 (18)
Sacubitril/Valsartan	7 (25)
MRA	12 (43)
SGLT-2 inhibitor	11 (39)
Statin	9 (32)
Anticoagulation	6 (21)

ACE: angiotensin-converting enzyme; ARB: angiotensin receptor blocker; CKD: chronic kidney disease; COPD: chronic obstructive airways disease; CV: cardiovascular; MRA: mineralocorticoid receptor antagonist; and SGLT-2: Sodium-glucose co-transporter-2. Continuous variables are displayed as mean ± SD or median (interquartile range). Categorical variables were displayed as number (%).

**Table 3:** Cardiovascular magnetic resonance (CMR) data of sepsis survivors.

	Patients (*n* = 28)
Day from sepsis event to CMR	65 (12–124)
Indications for CMR	
Abnormal echocardiography	
LV dysfunction	17 (61)
Pericardial effusion	4 (14)
Clinical symptoms alone	7 (25)
CMR volumes and function	
LV EDVi, mL/m^2^	94 (79–108)
LV ESVi, mL/m^2^	36 (28–57)
LV SVi, mL/m^2^	49 ± 13
LV EF, %	59 (43–64)
RV EDVi, mL/m^2^	83 ± 21
RV ESVi, mL/m^2^	39 (30–44)
RV SVi, mL/m^2^	44 ± 12
RV EF, %	53 (48–60)
LV mass index, g/m^2^	69 ± 19
LGE data	
LV LGE present	18 (64)
Non-ischaemic pattern	16 (57)
Ischaemic pattern	2 (7)
LGE as % LV mass, %	3.3 (0.9–7.1)
Patients with LVEF < 50%, %	7.3 ± 6.0
Patient with LVEF ≥ 50%, %	3.1 ± 2.5
RV LGE present	0 (0)

CMR: cardiovascular magnetic resonance; EDVi: end-diastolic volume index; EF: ejection fraction; ESVi: end-systolic volume index; LGE: late gadolinium enhancement; LV: left ventricular; RV: right ventricular; SVi: stroke volume index. Continuous variables are displayed as mean ± SD or median (interquartile range). Categorical variables were displayed as number (%).

**Table 4:** Myocardial fibrosis burden as assessed by LGE as stratified by co-morbidities.

	LGE as % of LV mass		
	With co-morbidity	Without co-morbidity	*p* Value
Atrial fibrillation	7.7 ± 6.3	4.1 ± 4.1	0.234
Hypertension	4.2 ± 5.7	5.1 ± 4.7	0.772
Diabetes mellitus	3.4 ± 3.3	5.1 ± 4.9	0.502
Hypercholesterolaemia	1.6 ± 1.4	5.5 ± 4.9	0.005
Chronic kidney disease	10.5 ± 5.3	4.5 ± 4.6	0.365
Composite of the above	5.7 ± 5.6	4.1 ± 3.8	0.411

LGE: late gadolinium enhancement; LV: left ventricular.

## Data Availability

The patient data in this study cannot be shared in the public domain; reasonable requests for the data should be sent to the corresponding author.

## References

[1] Angus D.C., van der Poll T. (2013). Severe Sepsis and Septic Shock. New Engl. J. Med..

[2] Prescott H.C., Langa K.M., Iwashyna T.J. (2015). Readmission Diagnoses After Hospitalization for Severe Sepsis and Other Acute Medical Conditions. JAMA.

[3] Jentzer J.C., Lawler P.R., Van Houten H.K., Yao X., Kashani K.B., Dunlay S.M. (2023). Cardiovascular Events Among Survivors of Sepsis Hospitalization: A Retrospective Cohort Analysis. J. Am. Hear. Assoc..

[4] Egi M., Ogura H., Yatabe T., Atagi K., Inoue S., Iba T., Kakihana Y., Kawasaki T., Kushimoto S., Kuroda Y. (2021). The Japanese Clinical Practice Guidelines for Management of Sepsis and Septic Shock 2020 (J-SSCG 2020). Acute Med. Surg..

[5] Evans L., Rhodes A., Alhazzani W., Antonelli M., Coopersmith C.M., French C., Machado F.R., Mcintyre L., Ostermann M., Prescott H.C. (2021). Surviving Sepsis Campaign: International Guidelines for Management of Sepsis and Septic Shock 2021. Crit. Care Med..

[6] Guarino M., Perna B., Cesaro A.E., Maritati M., Spampinato M.D., Contini C., De Giorgio R. (2023). 2023 Update on Sepsis and Septic Shock in Adult Patients: Management in the Emergency Department. J. Clin. Med..

[7] Parker M.M., Shelhamer J.H., Bacharach S.L., Green M.V., Natanson C., Frederick T.M., Damske B.A., Parrillo J.E. (1984). Profound but Reversible Myocardial Depression in Patients with Septic Shock. Ann. Intern. Med..

[8] Parker M.M., Suffredini A.F., Natanson C., Ognibene F.P., Shelhamer J.H., Parrillo J.E. (1989). Responses of left ventricular function in survivors and nonsurvivors of septic shock. J. Crit. Care.

[9] Ravikumar N., Sayed M.A., Poonsuph C.J., Sehgal R., Shirke M.M., Harky A. (2021). Septic Cardiomyopathy: From Basics to Management Choices. Curr. Probl. Cardiol..

[10] Boissier F., Razazi K., Seemann A., Bedet A., Thille A.W., de Prost N., Lim P., Brun-Buisson C., Dessap A.M. (2017). Left ventricular systolic dysfunction during septic shock: The role of loading conditions. Intensiv. Care Med..

[11] Hasegawa D., Ishisaka Y., Maeda T., Prasitlumkum N., Nishida K., Dugar S., Sato R. (2023). Prevalence and Prognosis of Sepsis-Induced Cardiomyopathy: A Systematic Review and Meta-Analysis. J. Intensiv. Care Med..

[12] Angriman F., Rosella L.C., Lawler P.R., Ko D.T., Wunsch H., Scales D.C. (2022). Sepsis hospitalization and risk of subsequent cardiovascular events in adults: A population-based matched cohort study. Intensiv. Care Med..

[13] Arens C., Bajwa S.A., Koch C., Siegler B.H., Schneck E., Hecker A., Weiterer S., Lichtenstern C., Weigand M.A., Uhle F. (2016). Sepsis-induced long-term immune paralysis—results of a descriptive, explorative study. Crit. Care.

[14] Mankowski R.T., Yende S., Angus D.C. (2018). Long-term impact of sepsis on cardiovascular health. Intensiv. Care Med..

[15] Davydow D.S., Hough C.L., Langa K.M., Iwashyna T.J. (2013). Symptoms of Depression in Survivors of Severe Sepsis: A Prospective Cohort Study of Older Americans. Am. J. Geriatr. Psychiatry.

[16] Calsavara A.J., Nobre V., Barichello T., Teixeira A.L. (2018). Post-sepsis cognitive impairment and associated risk factors: A systematic review. Aust. Crit. Care.

[17] Paoli C.J., Reynolds M.A., Sinha M., Gitlin M., Crouser E. (2018). Epidemiology and Costs of Sepsis in the United States—An Analysis Based on Timing of Diagnosis and Severity Level*. Crit. Care Med..

[18] Malomo S., Oswald T., Stephenson E., Yip A., Alway T., Hadjivassilev S., Coombs S., Ellery S., Lee J., James R. (2025). Characterisation of Post-Sepsis Cardiomyopathy Using Cardiovascular Magnetic Resonance. Diagnostics.

[19] Oswald T., Malomo S., Alway T., Hadjivassilev S., Coombs S., Ellery S., Lee J., Phillips C., Philips B., James R. (2025). Guideline-Directed Medical Therapy in Sepsis Survivors with Left Ventricular Systolic Dysfunction: An Observational Study. J. Clin. Med..

[20] Schmittinger C.A., Dünser M.W., Torgersen C., Luckner G., Lorenz I., Schmid S., Joannidis M., Moser P., Hasibeder W.R., Halabi M. (2013). Histologic Pathologies of the Myocardium in Septic Shock. Shock.

[21] Hundley W.G., Bluemke D.A., Bogaert J., Flamm S.D., Fontana M., Friedrich M.G., Grosse-Wortmann L., Karamitsos T.D., Kramer C.M., Kwong R.Y. (2022). Society for Cardiovascular Magnetic Resonance (SCMR) guidelines for reporting cardiovascular magnetic resonance examinations. J. Cardiovasc. Magn. Reson..

[22] Messroghli D.R., Moon J.C., Ferreira V.M., Grosse-Wortmann L., He T., Kellman P., Mascherbauer J., Nezafat R., Salerno M., Schelbert E.B. (2016). Clinical recommendations for cardiovascular magnetic resonance mapping of T1, T2, T2* and extracellular volume: A consensus statement by the Society for Cardiovascular Magnetic Resonance (SCMR) endorsed by the European Association for Cardiovascular Imaging (EACVI). J. Cardiovasc. Magn. Reson..

[23] Benz D.C., Gräni C., Antiochos P., Heydari B., Gissler M.C., Ge Y., Cuddy S.A.M., Dorbala S., Kwong R.Y. (2023). Cardiac magnetic resonance biomarkers as surrogate endpoints in cardiovascular trials for myocardial diseases. Eur. Hear. J..

[24] Antiochos P., Ge Y., Steel K., Bingham S., Abdullah S., Mikolich J.R., Arai A.E., Bandettini W.P., Patel A.R., Farzaneh-Far A. (2020). Imaging of Clinically Unrecognized Myocardial Fibrosis in Patients With Suspected Coronary Artery Disease. Circ..

[25] Aquaro G.D., De Gori C., Faggioni L., Parisella M.L., Cioni D., Lencioni R., Neri E. (2023). Diagnostic and prognostic role of late gadolinium enhancement in cardiomyopathies. Eur. Hear. J. Suppl..

[26] Trimarchi G., Teresi L., Licordari R., Pingitore A., Pizzino F., Grimaldi P., Calabrò D., Liotta P., Micari A., de Gregorio C. (2024). Transient Left Ventricular Dysfunction from Cardiomyopathies to Myocardial Viability: When and Why Cardiac Function Recovers. Biomedicines.

[27] Kali A., Choi E.-Y., Sharif B., Kim Y.J., Bi X., Spottiswoode B., Cokic I., Yang H.-J., Tighiouart M., Conte A.H. (2015). Native T 1 Mapping by 3-T CMR Imaging for Characterization of Chronic Myocardial Infarctions. JACC: Cardiovasc. Imaging.

[28] Mikami Y., Cornhill A., Heydari B., Joncas S.X., Almehmadi F., Zahrani M., Bokhari M., Stirrat J., Yee R., Merchant N. (2016). Objective criteria for septal fibrosis in non-ischemic dilated cardiomyopathy: Validation for the prediction of future cardiovascular events. J. Cardiovasc. Magn. Reson..

[29] McDonagh T.A., Metra M., Adamo M., Gardner R.S., Baumbach A., Böhm M., Burri H., Butler J., Čelutkienė J., Chioncel O. (2021). 2021 ESC Guidelines for the diagnosis and treatment of acute and chronic heart failure. Eur Heart J..

[30] Malomo S., Oswald T., Alway T., Hadjivassilev S., Coombs S., Ellery S., Lee J., Phillips C., Philips B., James R. (2025). Characterization of Coronary Artery Disease in Sepsis Survivors. Biomedicines.

[31] Meier C., Eisenblätter M., Gielen S. (2024). Myocardial Late Gadolinium Enhancement (LGE) in Cardiac Magnetic Resonance Imaging (CMR)—An Important Risk Marker for Cardiac Disease. J. Cardiovasc. Dev. Dis..

[32] Kim R.J., Wu E., Rafael A., Chen E.-L., Parker M.A., Simonetti O., Klocke F.J., Bonow R.O., Judd R.M. (2000). The Use of Contrast-Enhanced Magnetic Resonance Imaging to Identify Reversible Myocardial Dysfunction. New Engl. J. Med..

[33] Fouda S., Godfrey R., Pavitt C., Alway T., Coombs S., Ellery S.M., Parish V., Silberbauer J., Liu A. (2025). Cardiac Sarcoidosis and Inherited Cardiomyopathies: Clinical Masquerade or Overlap?. J. Clin. Med..

[34] Liu A., Munemo L.T., Martins N., Kouranos V., Wells A.U., Sharma R.K., Wechalekar K. (2025). Assessment of Cardiac Sarcoidosis with PET/CT. J. Nucl. Med. Technol..

[35] Messroghli D.R., Walters K., Plein S., Sparrow P., Friedrich M.G., Ridgway J.P., Sivananthan M.U. (2007). Myocardial *T*_1_ mapping: Application to patients with acute and chronic myocardial infarction. Magn. Reson. Med..

[36] Dabir D., Child N., Kalra A., Rogers T., Gebker R., Jabbour A., Plein S., Yu C.-Y., Otton J., Kidambi A. (2014). Reference values for healthy human myocardium using a T1 mapping methodology: Results from the International T1 Multicenter cardiovascular magnetic resonance study. J. Cardiovasc. Magn. Reson..

[37] Puntmann V.O., Carerj M.L., Wieters I., Fahim M., Arendt C., Hoffmann J., Shchendrygina A., Escher F., Vasa-Nicotera M., Zeiher A.M. (2020). Outcomes of Cardiovascular Magnetic Resonance Imaging in Patients Recently Recovered From Coronavirus Disease 2019 (COVID-19). JAMA Cardiol..

[38] Hiraiwa H., Kasugai D., Okumura T., Murohara T. (2024). Clinical implications of septic cardiomyopathy: A narrative review. Medicine.

[39] Kakihana Y., Ito T., Nakahara M., Yamaguchi K., Yasuda T. (2016). Sepsis-induced myocardial dysfunction: Pathophysiology and management. J. Intensiv. Care.

[40] L’hEureux M., Sternberg M., Brath L., Turlington J., Kashiouris M.G. (2020). Sepsis-Induced Cardiomyopathy: A Comprehensive Review. Curr. Cardiol. Rep..

[41] Sato R., Nasu M. (2015). A review of sepsis-induced cardiomyopathy. J. Intensiv. Care.

[42] Oswald T., Coombs S., Ellery S., Liu A. (2025). Now and the Future: Medications Changing the Landscape of Cardiovascular Disease and Heart Failure Management. J. Clin. Med..

